# Supplementary material for: Evolutionary and functional history of the Escherichia coli K1 capsule
Source: Nat Commun. 2023 Jun 15;14:3294. doi: 10.1038/s41467-023-39052-w (PMC10272209; doi:10.1038/s41467-023-39052-w)
Supplement: Supplementary file 1 — Supplementary Information [file 41467_2023_39052_MOESM1_ESM.pdf]

Supplementary Information

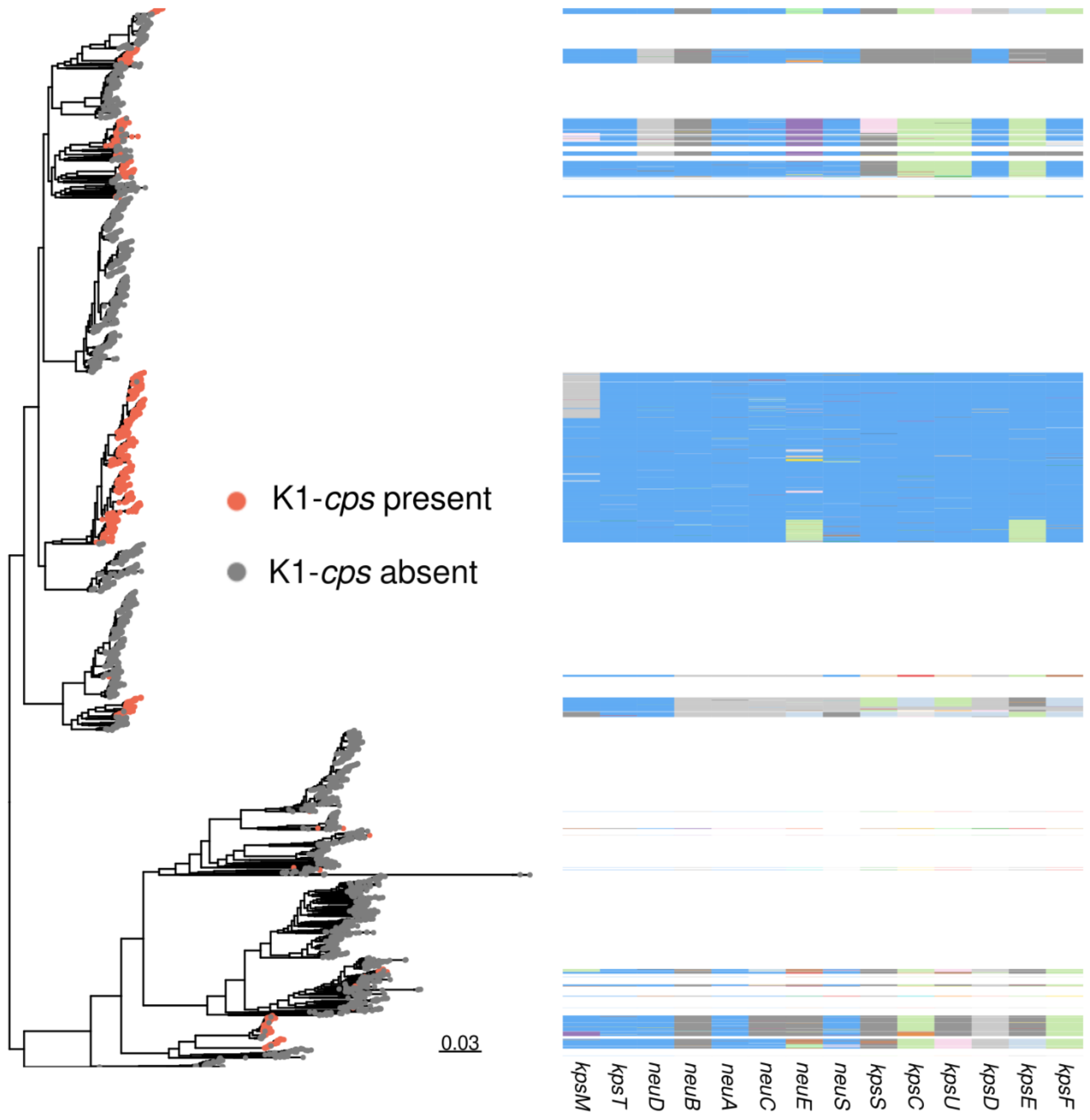

Supplementary Figure 1. Variation in the amino acid sequences of the 14 genes belonging to the K1-*cps* locus. For each sequence, the different allelic variants (any different amino acid per position) are represented with a distinct colour. The phylogeny on the left corresponds to the maximum-likelihood tree described in Figure 1B with tips coloured based on the absence (in grey) or presence (in red) of the K1-*cps* locus.

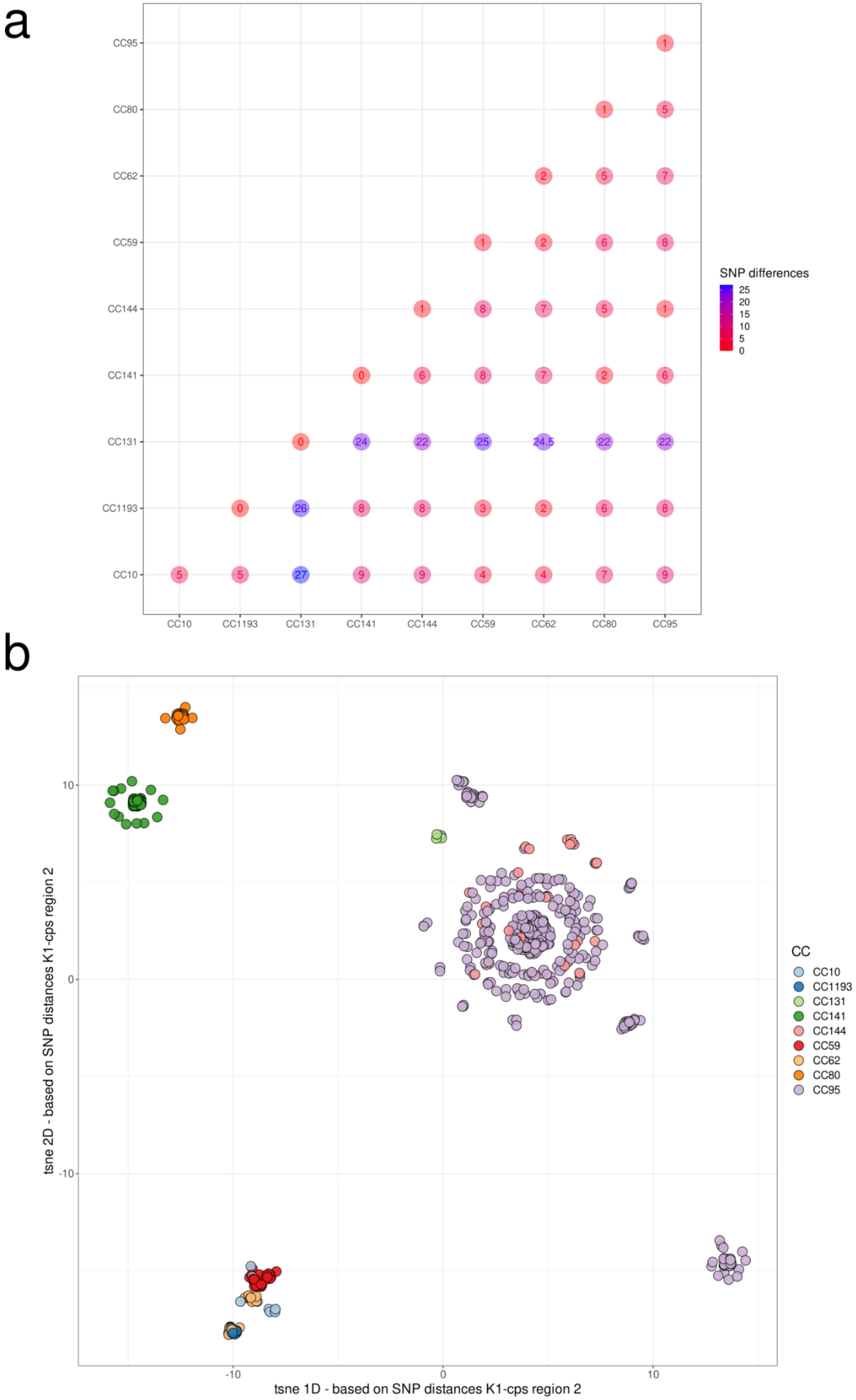
a
CC95
CC80
CC62
CC59
CC144
CC141
CC131
CC1193
CC10
CC10
CC1193
CC131
CC141
CC144
CC59
CC62
CC80
CC95
SNP differences
25
20
15
10
5
0
b
tsne 2D - based on SNP distances K1-cps region 2
tsne 1D - based on SNP distances K1-cps region 2
10
0
-10
-10
0
10
CC
CC10
CC1193
CC131
CC141
CC144
CC59
CC62
CC80
CC95

Supplementary Figure 2. a) The median number of SNP differences for region 2 of the K1-*cps* locus (*neu* genes). Isolates belonging to the same lineage (in the diagonal) tend to have a lower number of SNP differences compared to isolates between distinct lineages. **b)** The SNP distance matrix of region 2 of the K1-*cps* locus was embedded into two dimensions using t-distributed stochastic neighbor embedding (tsne). In the plot, each circle corresponds to a single isolate and its colour corresponds to the clonal complex (CC) lineage. For visualization purposes, we added a small amount of random variation in each point to avoid overplotting of the isolates with an identical SNP profile.

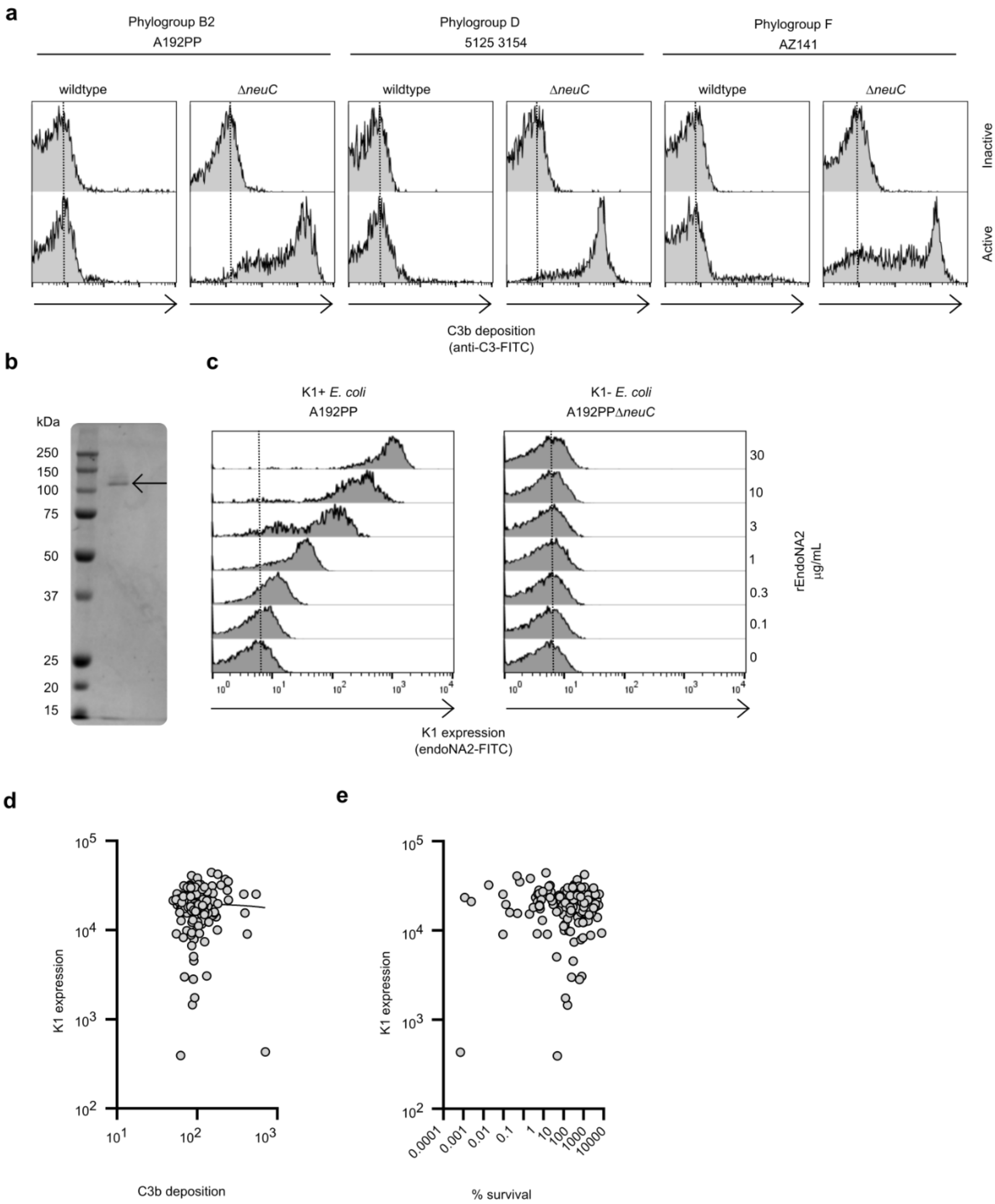

Supplementary Figure 3. a) K1 capsule expression enables *E. coli* strains to resist complement C3b deposition by human serum. Representative flow cytometry plots show total C3b deposition on the surface of isogenic *E. coli* strains following a 30-minute incubation in human serum (AS) or heat-inactivated serum (HIS; inactive serum) at 37ºC. **b)** Expression and purification of recombinant (r)EndoNA2-GFP from a *E. coli* expression system. An arrow signals the proteins of expected size. Representative of two images. **c)** Binding of varying concentrations of rEndoNA2-GFP to isogenic K1+ and K1- *E. coli* strains, measured by flow cytometry analysis. **d)** Relationship between K1 expression and C3b deposition. Each data point represents a single *E. coli* isolate. **e)** Relationship between K1 expression and serum survival. Each data point represents a single *E. coli* isolate.

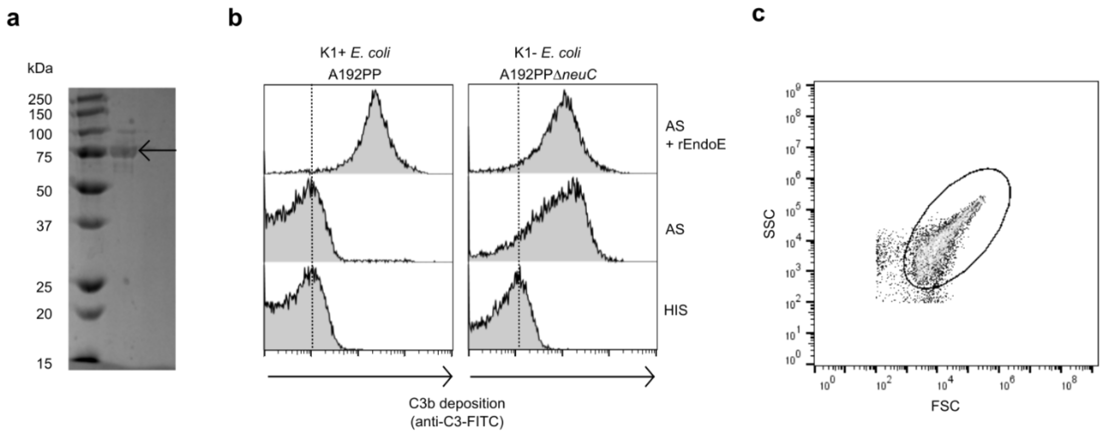

Supplementary Figure 4. a) Expression and purification of recombinant (r)EndoE from an *E. coli* expression system. An arrow signals the proteins of expected size. Representative of two images. **b)** The effect of rEndoE capsule depolymerase on deposition of complement C3b on isogenic K1+ and K1- *E. coli* strains (background A192PP) after incubation in human active serum.
