# Supplementary material for: Evolutionary and functional history of the Escherichia coli K1 capsule
Source: Nat Commun. 2023 Jun 15;14:3294. doi: 10.1038/s41467-023-39052-w (PMC10272209; doi:10.1038/s41467-023-39052-w)
Supplement: Supplementary file 3 — Description of Additional Supplementary Files [file 41467_2023_39052_MOESM3_ESM.pdf]

Description of Additional Supplementary Files

Filename: **Supplementary Dataset S1**

Description: Table with the metadata from the 5,065 genomes representing the global collection of ExPEC isolates used to assess the presence of the K1-*cps* locus. The accession column provides the reference to the short-read data publicly available in the ENA database.

Filename: **Supplementary Dataset S2**

Description: Table with the statistics behind the BacDating models used to compute the dating phylogenies from the main clonal complexes presented in the manuscript. MCMC stands for Markov Chain Monte Carlo. ESS stands for the estimated sample size of the parameters (mu, sigma, alpha) which was used to assess the convergence of the parameters in the MCMC. DIC stands for deviance information criterion which was used to compare the resulting BactDating models against a model with fixed equal dates and assess the significance of the temporal signal. Gelman-Rubins refers to the convergence diagnostic used to assess the convergence of the parameters in the MCMC.

Filename: **Supplementary Dataset S3**

Description: Table with the 44 genome accessions corresponding to complete genomes from CC95 which were retrieved from the RefSeq database. These genomes were used to infer the position of the K1-*cps* locus in the *E. coli* genome and characterise the gene synteny present upstream and downstream of the locus (presence of the pathogenicity island). The isolation source and their associated host are also indicated in the table.

Filename: **Supplementary Dataset S4**

Description: Table with the 9 complete chromosomal sequences from the NORM collection generated in this study corresponding to the main clonal lineages having the K1-*cps* locus. For each genome, we provide the accession to their associated short- and long-read data and provide a permanent figshare link to make their chromosomal sequences available.

Filename: **Supplementary Dataset S5**

Description: Table detailing primers and plasmids used in the construction of Δ*neuC* isogenic mutant strains.

Filename: **Supplementary Dataset S6**

Description: Table with the isogenic *E. coli* strains used in the study. For each strain, we provide the phylogroup, clonal complex (CC), sequence type (ST) and generation approach.

Filename: **Supplementary Dataset S7**
Description: Table with the 149 isolates used in the study in population-based assays in Fig 5d, e and f. For each strain, we provide the phylogroup, clonal complex (CC) and sequence type (ST).

Filename: **Supplementary Dataset S8**
Description: Table with the 22 isolates used in the study in rEndoE-based assays in Fig 6c and d. For each strain, we provide the phylogroup, clonal complex (CC) and sequence type (ST).
